# Daily Rhythm of Fractal Cardiac Dynamics Links to Weight Loss Resistance: Interaction with *CLOCK* 3111T/C Genetic Variant

**DOI:** 10.3390/nu13072463

**Published:** 2021-07-19

**Authors:** Hui-Wen Yang, Marta Garaulet, Peng Li, Cristina Bandin, Chen Lin, Men-Tzung Lo, Kun Hu

**Affiliations:** 1 Graduate Institute of Communication Engineering, National Taiwan University, Taipei 10617, Taiwan; hyang39@bwh.harvard.edu; 2 Institute of Translational and Interdisciplinary Medicine, Department of Biomedical Sciences and Engineering, National Central University, Taoyuan 320317, Taiwan; clin@ncu.edu.tw; 3 Division of Sleep and Circadian Disorders, Department of Medicine and Neurology, Brigham and Women’s Hospital, Boston, MA 02115, USA; pli9@bwh.harvard.edu; 4 Division of Sleep Medicine, Department of Medicine, Harvard Medical School, Boston, MA 02115, USA; 5 Department of Physiology, University of Murcia, IMIB, 30071 Murcia, Spain; mcbandin@gmail.com

**Keywords:** circadian rhythm, dietary intervention, obesity treatment, weight control, autonomic function, fractal cardiac dynamics

## Abstract

The effectiveness of weight loss treatment displays dramatic inter-individual variabilities, even with well-controlled energy intake/expenditure. This study aimed to determine the association between daily rhythms of cardiac autonomic control and weight loss efficiency and to explore the potential relevance to weight loss resistance in humans carrying the genetic variant C at *CLOCK* 3111T/C. A total of 39 overweight/obese Caucasian women (20 *CLOCK* 3111C carriers and 19 non-carriers) completed a behaviour–dietary obesity treatment of ~20 weeks, during which body weight was assessed weekly. Ambulatory electrocardiographic data were continuously collected for up to 3.5 days and used to quantify the daily rhythm of fractal cardiac dynamics (FCD), a non-linear measure of autonomic function. FCD showed a 24 h rhythm (*p* < 0.001). Independent of energy intake and physical activity level, faster weight loss was observed in individuals with the phase (peak) of the rhythm between ~2–8 p.m. and with a larger amplitude. Interestingly, the phase effect was significant only in C carriers (*p* = 0.008), while the amplitude effect was only significant in TT carriers (*p* < 0.0001). The daily rhythm of FCD and *CLOCK* 3111T/C genotype is linked to weight loss response interactively, suggesting complex interactions between the genetics of the circadian clock, the daily rhythm of autonomic control, and energy balance control.

## 1. Introduction

Obesity is a major health problem, and effective strategies for body weight control are required to reduce the prevalence of obesity [1,2,3]. Indeed, one of the most common goals to attend to a nutritional clinic is to lose weight. One puzzling phenomenon is the dramatic inter-individual variability in response to obesity treatment. In weight loss therapies based on nutritional advice and behavioural–dietary changes, some individuals may lose up to 30% of their initial body weight, while some others may gain up to 10% of initial body weight [4,5]. Consistently, there is a large inter-individual variability in weight loss evolution during the treatment [6] such as weight loss speed (WLS; i.e., weight loss per week)—a key outcome measure that is linked to the drop-out rate during the treatments and predicts remission status in obesity treatments [7]. Understanding the factors contributing to WLS and related underlying mechanisms is one of the major tasks in obesity treatment [8].

Studies in chronobiology have established that physiological processes/functions display circadian rhythms in sync with the day–night cycle and these 24 h rhythms are generated and orchestrated by the circadian timing system [9,10]. There is ample evidence that disrupting the circadian rhythms have many adverse consequences including risk for obesity [11,12] and cardiometabolic disorders [13,14]. Specifically, more recent studies have shown that the timing of daily behaviours such as meals and exercise is important for body weight control [15,16,17]. Thus, it is believed that chronobiology may provide novel tools for obesity treatment. Supporting this, studies have revealed compelling evidence that the genetic influences on obesity are related to the circadian system. For instance, Circadian Locomotor Output Cycles Kaput (*CLOCK*), one of the transcription factors from the positive limb of the molecular clock in the primary feedback loop that generates circadian rhythms [18], is of great relevance to obesity [19,20]. In addition, our previous study suggested that *CLOCK* genes may also play a role in weight loss efficiency. The genetic variation in the rs1801260, named *CLOCK* 3111T/C SNP, has been associated with weight loss resistance, i.e., *CLOCK* 3111C carriers have more difficulties in losing weight than non-carriers [21]. The physiological/pathological pathways through which this genetic variant impacts weight loss are yet to be elucidated.

One of the common mechanistic pathways through which the circadian system and the behavioural cycles impact body weight control is the autonomic nervous system (ANS). This pathway is supported by the findings that (1) the ANS activity is modulated by the endogenous circadian rhythms [22,23,24]; (2) the ANS activity impacts metabolism [25] while responding to body weight change [26]; and (3) ANS dysfunction plays a crucial role in the pathophysiology of obesity [27]. However, how the circadian rhythm of autonomic function is linked to weight loss efficiency is not well studied.

ANS function can be non-invasively assessed from the patterns in heartbeat fluctuations that are controlled by the sympathetic and parasympathetic nervous systems. One of the non-invasive measures of cardiac autonomic control is the fractal patterns in heartbeat fluctuations or fractal cardiac dynamics (FCD), i.e., temporal structures or properties in the fluctuations that persist at different time scales [28]. FCD reflect a delicate dynamic interplay between sympathetic and vagal outflows [29], revealing the integrative autonomic control [30]. Fractal heartbeat fluctuations are altered under different physiological and pathological conditions such as wake/sleep stages and circadian time [22,31] and have been used in clinical studies to predict survival and mortality of patients after stroke or myocardial infarction [32,33,34]. Previous studies have shown that FCD are affected by daily behavioural cycles/activities, the circadian rhythms, and their interactions. For instance, FCD are reduced during sleep compared to during wakefulness [30,31]. Furthermore, FCD during wakefulness also display an endogenous circadian rhythm with a peak value at the circadian phase corresponding to 9–11 a.m. [22], and circadian misalignment (disturbed alignment between the circadian clock and the daily behavioural cycle)—the key biological disruption underlying the adverse effects of shift work—leads to increased FCD across the 24 h cycle [35]. As circadian misalignment is linked to the risk for obesity, the goal of the current study is to examine the potential role of the circadian regulation of FCD in the response to weight loss treatment, which may provide a useful tool or target in the context of precision nutrition therapy for better design of personalized obesity treatments. Here, we hypothesize that the daily rhythm of fractal cardiac dynamics varies among individuals and that differences in the timing and amplitude of the rhythm are linked to inter-individual differences in weight loss responses to obesity treatment. We also hypothesize that the daily rhythm of fractal cardiac dynamics differs between carriers of the risk variant (C) and non-carriers (TT), associating with the difficulty to lose weight in C carriers.

## 2. Materials and Methods

### 2.1. Participants

We performed a case-control study to examine the effects of the *CLOCK* 3111T/C variant and the daily rhythm of fractal heartbeat fluctuations on WLS. We studied the participants of the ONTIME (Obesity, Nutrigenomics, Time, Mediterranean) project who attended outpatient obesity clinics in the city of Murcia (Spain) for a dietary obesity treatment. The original protocol of the ONTIME project has been previously published [36]. In this study, 40 overweight/obese Caucasian women (BMI > 25) were recruited, with 20 C carriers (including 3 CC carriers and 17 TC carriers) and 20 TT carriers in *CLOCK* 3111T/C using the dominant model. The selection of the dominant model (i.e., combining CC and TC) was based on previous studies where we have tested different genetic inheritance models for obesity and weight loss and a dominant model was applied in the final analyses for *CLOCK* 3111T/C [21,37]. Those carriers of the risk variant C were first randomly chosen from the previously established database of the ONTIME project who participated in the weight loss program within the past 3 months, and then 20 TT carriers were chosen to match the age, obesity parameters, energy intake and expenditure, and menopausal status of the 20 C carriers (Table 1). One TT carrier discontinued participation during the study. No participants received thermogenic, lipogenic, or sleep drugs or melatonin. All participants were free of the following diseases/disorders: insomnia, cognitive disorders, diabetes mellitus, chronic renal failure, hepatic diseases, and cancer. Participants provided a written informed consent prior to enrolment in the study. The study was carried out in accordance with good clinical practice and was approved by the Ethical Committee of the University of Murcia (Clinicaltrials.gov #NCT02829619). Note that, in the trial register of the ONTIME study, daily rhythms of autonomic function assessed by ambulatory electrocardiography were included as one of the secondary outcome measures. However, the specific analyses or outcome variables for the daily rhythms of autonomic function such as fractal cardiac dynamics were not available in the trial. Thus, the current study involves an exploratory post hoc analysis of a retrospectively registered case control study.

### 2.2. Weight Loss Treatment 

The weight loss intervention program has been published previously [5]. In brief, participants attended weekly 60 min group therapy sessions. Each session was conducted by a nutritionist and consisted of the following four components: (1) dietary advice, based on the principles of the Mediterranean diet, in which the distribution of macronutrient followed the recommendations of the Spanish Society of Community Nutrition [38]; (2) nutritional education; (3) moderate increase in physical activity; and (4) cognitive–behavioural modifications, including *stimulus* control, self-monitoring, positive reinforcement, preventing relapse, and cognitive restructuring. The duration of the program depended on the individual weight loss goal (mean duration of ~20 weeks).

### 2.3. Weight Loss Evolution

During the treatment, body weight was measured weekly when participants attended the therapy sessions. Accumulated weight loss at each week (i.e., difference from the initial weight) was expressed as a percentage of the initial weight. 

### 2.4. Physical Activity and Energy Intake Assessment

Dietary intake was assessed using a single 24 h dietary recall where participants indicated type, quantity, and preparation of each recorded eating episode. Recalls were conducted from Monday to Friday, thus capturing the information on both weekdays (from Monday to Thursday) and weekends (Sunday). Energy intakes per 24 h and per meal as well as macronutrient composition were determined using the nutritional evaluation software program Nutrilet, based on Spanish food composition tables [39].

In addition, participants completed the International Physical Activity Questionnaire that assesses physical activity over the past 7 days. This questionnaire has been previously validated in a Spanish population, and good correlation was obtained with accelerometer-derived physical activity estimates [40,41]. A total activity score capturing intensity and timing was calculated as Metabolic Units (METs) in minutes per week.

### 2.5. DNA Isolation and CLOCK Genotyping

DNA was isolated from blood samples using standard procedures (Qiagen, Valencia, CA, USA). We performed genotyping of the *CLOCK* 3111T/C SNP using a TaqMan assay with allele-specific probes on the ABI Prism 7900HT Sequence Detection System (Applied Biosystems, Foster City, CA, USA) according to the standardized laboratory protocols previously used for this genetic variant [20].

### 2.6. Ambulatory Electrogradiography (ECG) and Heartbeat Recordings

After finishing the weight loss program, participants visited the clinic where procedures to place/remove the ECG electrodes were demonstrated by the research team. After the visit, each participant brought home an ECG recorder, electrodes, and written instructions. Following the guidelines for heartrate variability analysis published by The European Society of Cardiology and The North American Society of Pacing and Electrophysiology [42], ambulatory 4-lead ECG data were continuously collected in each participant for up to 3.5 days at a sampling frequency of 1000 Hz using a Holter monitor (MyECG E3-80, Mircostar Company, Taipei, Taiwan; PhysiolGuard, Taipei, Taiwan). During the ambulatory study phase, participants continuously wore the device except when showering. To obtain heartbeat intervals, R waves in the ECG recordings were first identified by the published QRS detection program [43] and then were visually verified by a trained technician to ensure that only normal sinus R waves were included for further analysis.

### 2.7. Fractal Analysis of Heartbeat Fluctuations

To assess fractal cardiac dynamic (FCD), detrended fluctuation analysis (DFA) [44] was performed to examine temporal correlations in heartbeat fluctuations (i.e., whether heartbeat intervals were random or the current heartbeat values affect the following values due to the physiological feedbacks) at different time scales. For a signal with fractal fluctuations, DFA provides a scaling exponent, α, that quantifies the temporal correlation: α = 0.5 indicates no correlation and the signal is random, and α > 0.5 indicates positive correlations (i.e., large values of heartbeat intervals are more likely followed by large values of heartbeat intervals, and vice versa). For instance, α at time scales >30 heartbeats becomes close to 0.5 for patients with atrial fibrillation [45].

DFA includes the following steps: (i) In each 1 h heartbeat series, the signal was integrated after removing the 1 h mean. (ii) For a selected time scale n (in the unit of the number of heartbeats), the integrated signal was divided into non-overlapping windows each with n beats. (iii) In each window, the local trend was determined by fitting the data with a quadratic function. (iv) The fluctuation amplitude *F(n)* at the scale *n* was the root–mean–square of all the residuals after removing the local trends in all windows. (v) The fluctuation function *F(n)* was obtained by repeating (ii)–(iv) for different values of time scale n. Fractal patterns can be characterized by a power–law function of *F(n)*, i.e., $F\left(n\right)\approx n^{\alpha }$ where α is the scaling exponent, estimating the temporal correlation. The α at time scales between 70 and 300 beats was used in this study, because the fractal correlations of heartbeat fluctuations in healthy subjects is robust within this time scale region [46] and the correlation is regulated by the sleep–wake cycle and the circadian rhythm [22,31].

### 2.8. Rhythmicity Assessment

To quantify the daily rhythm of FCD (i.e., daily α rhythm) in each subject, we considered the amplitude and phase of the 24 h α rhythm. Specifically, the hourly α values were fitted using a cosinor analysis with a 24 h sinusoidal function and a 12 h harmonic, i.e., $\alpha \left(t\right)=\mu +A\cos\left(2\pi t/24-\varphi \right)+B\cos(2\pi t/12-\theta )+\varepsilon$, where μ is the mean, and ε represents residual error, $A\cos\left(2\pi t/24-\varphi \right)=A_{1}\cos\left(2\pi t/24\right)+A_{2}\sin\left(2\pi t/24\right),$ and $B\cos\left(2\pi t/12-\theta \right)=B_{1}\cos\left(2\pi t/12\right)+B_{2}\sin\left(2\pi t/12\right)$. The coefficient of the 24 h component in the cosinor fit, $A=\sqrt{A_{1}^{2}+A_{2}^{2}}$, was used to estimate the amplitude of the 24 h rhythm, and ${\varphi =\tan}^{-1}\left(A_{2}/A_{1}\right)$ was the phase representing the peak time of the 24 h rhythm. The rhythm was determined to be not significant if both *p*-values for $A_{1}$ and $A_{2}$ are >0.05. In this case, the subject is removed from the analysis related to the effect of phase on weight loss, because the phase estimation might be unreliable.

### 2.9. Statistical Analysis

To assess the association between the daily rhythm of FCD and the effectiveness of the weight loss intervention, two linear mixed effect models were performed with the accumulative weight loss percentage (of the initial body weight) as the outcome. Model 1a: the treatment time (weeks from baseline) and its interaction with the amplitude and phase of the 24 h α rhythm, respectively, are predictors in the model. Subject-specific intercept were included as a random factor. The coefficient of the treatment time quantifies the mean weight loss speed (WLS: a measure of weight loss effectiveness of the intervention), and the coefficients of the interactions between treatment week and amplitude/phase represent the effects of the 24 h α rhythm on WLS. *Model 1b*: as a supplementary analysis, daily energy intake and physical activity level, and their interaction with time were added to Model 1a as the predictors to test whether the effects of the 24 h α rhythm exist after controlling for energy intake and expenditure.

To test the difference in the daily rhythm of FCD between C carriers and non-carriers, *t*-tests were performed to compare the genetic differences in the amplitude and, separately, the acrophase. Additionally, in order to increase the power to access the group difference in the daily α rhythm, we also used a mixed model in which the cosinor fitting was performed on the pooled hourly α values of all 39 subjects as the outcome measures; the 24 h sinusoidal functions, genotype group (TC/CC and TT), and their interactions were included as fixed factors, and subject was a random effect on the MESOR/intercept. A bootstrap approach was used to determine whether the group difference in the 24 h profile was due to the differences in the amplitude and/or phase of the 24 h rhythm. Detailed methods were described in our previous study [47].

To examine whether the alterations in the daily α rhythm underlie the genetic effect on WLS, we compared the effect of *CLOCK* 3111T/C genotype on WLS with and without adjusting for the daily α rhythm. We first performed a simple linear mixed effect model (*Model 2a*) in which the accumulative weight loss percentage at each week as the outcome, the treatment time, and the interaction of treatment time and *CLOCK* 3111T/C genotype (TT or C carrier) were included as predictors, and subject was the random intercept. Then, in the secondary model (*Model 2b*), the amplitude and phase of the 24 h α rhythm and their interaction with treatment time were added to the model. If the effect of the genotype on WLS was diminished or reduced in Model 2b as compared to that in *Model 2a*, the daily rhythm should underlie or at least partially explain the genetic effect.

The third model (*Model 3*) was used to explore the potential interaction between the effects of genetics and the 24 h α rhythm on WLS. To achieve this, the following 3-way interactions were added to Model 2b as predictors: (1) the interaction of the amplitude of the 24 h α rhythm, *CLOCK* 3111T/C genotype, and treatment time; and (2) the interaction of phase of the 24 h α rhythm, *CLOCK* 3111T/C genotype, and time.

All variables in the models were continuous except for the genetic group and the phase of the 24 h α rhythm. Because the individual phase values were not continuously distributed and displayed separate peaks (Figure S1a [48]), we included the phase as a categorized variable with three groups: (1) “very late phase group” (12 a.m.–4 a.m.); (2) “late phase group” (2:40 p.m.–8 p.m.); and (3) “early phase group” (10 a.m.–2:40 p.m.), where the early and late phase were divided by the median value of the phases withing the range 10 a.m.–8 p.m. In all the models, if the interaction with treatment week was significant, post hoc analyses were performed to identify (1) the differences between the three phase groups and/or (2) the differences between the genetic group. In Model 3, if the 3-way interaction of amplitude/phase, genotype, and treatment time were significant, we further divided the data by the genetic group and tested the effect of amplitude and phase on treatment week separately. All statistical analyses were conducted in R (v. 4.0.2) with packages “lme4” and “lmerTest” for the linear mixed models, and packages “emmeans” and “interactions” for the post hoc analysis.

## 3. Results

### 3.1. Large Inter-Individual Variability in Weight Loss Effectiveness

**Figure 1:**
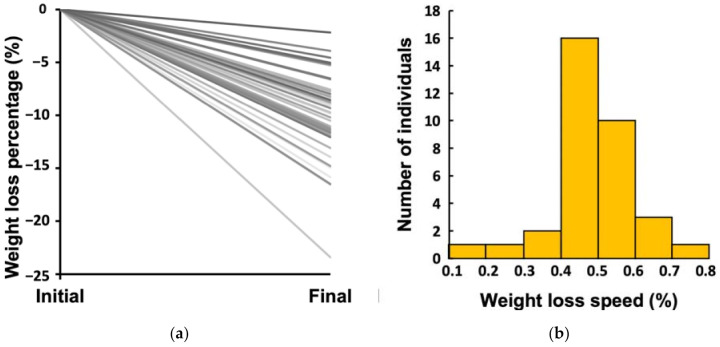
Inter-individual variability in the response to a weight loss treatment: (**a**) total weight loss percentage; (**b**) average weekly weight loss percentage or weight loss speed (WLS) during the treatment.

The average total weight loss percentage from initial body weight was 10.10%, and there was a high inter-individual variability (range: 2.16−23.41% from initial body weight) (Figure 1a). In total, 18 participants lost more than 10% of their initial body weight, while 21 participants lost <10%, including 3 participants who lost less than 5%. The weekly weight loss percentage or WLS was 0.44%, on average, which was equivalent to 334 g per week for an initial body weight of 76 kg (the group mean). WLS also displayed a high inter-individual variability with a range of 0.19−0.71% (equivalent to 144–540 g per week for an initial body weight of 76 kg) (Figure 1b).

### 3.2. Daily Rhythm of FCD and Large Inter-Individual Variations

**Figure 2:**
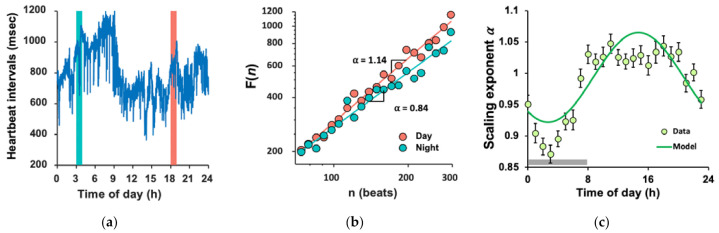
Daily rhythm of FCD: (**a**) A sample recording of 1-day heartbeat intervals. Data were collected from a 39-year-old woman not carrying *CLOCK* 3111 C allele. (**b**) Representative detrended fluctuation functions, *F*(*n*), of the two 1 h recordings marked in A (pink for the daytime; and cyan for the night-time). Data are shown on log–log plots. On the abscissa, *n* presents the time scale in heartbeats. The detrended fluctuation functions *F*(*n*) are vertically shifted for better visualization of the difference in the scaling exponent α (slope) between the daytime and night-time; (**c**) Mean daily rhythm of the scaling exponent α in all 39 subjects and the 24 h rhythm fitted by cosinor analysis. Error bars indicate standard error of the mean. The grey horizontal line indicates the average period of sleep for all participants.

Heartbeat fluctuations exhibited fractal patterns as indicated by a power–law fluctuation function (Figure 2b). The mean α across 24 h was 0.983 ± 0.003 (SE), indicating strong temporal correlations in the fluctuations (note α = 0.5 for white noise).

The α value showed a 24 h rhythm with lower values (i.e., more random heartbeat fluctuations) during the night-time and higher values (i.e., more correlated heartbeat fluctuations) during the daytime (Figure 2c). The cosinor analysis of individual data showed that the 24 h rhythm was significant in all subjects except for 3 participants (36 out of 39) (*p* < 0.05). Both the phase (φ) and amplitude (A) of the 24 h α rhythm showed large inter-individual differences. For most participants, the phase was during the daytime (10 a.m.–8 p.m.). In contrast, four participants had their peaks after midnight or during early morning, with two in each of the genetic groups (Figure S1a [48]). Amplitude ranged between 0.02 and 0.18 (median = 0.088; Mean ± SD = 0.09 ± 0.04); the largest amplitude was nine times that of the lowest (Figure S1b [48]).

### 3.3. Participants with Larger Amplitude and the Later Phase of the Daily α Rhythm Lost More Weight

*Model 1a* revealed a positive association between the amplitude of the daily α rhythm and weight loss (*p* < 0.001 for the interaction between treatment time and 24 h α amplitude, Supplementary Table S1 [48]). Specifically, an increase of 0.04 (1 SD of all the individual values) in the 24 h α amplitude was associated with an increase of ~0.05% (of the initial body weight, which is equivalent to 38 g for a 76 kg woman (average weight at baseline) per week in weight loss. The phase of the daily α rhythm was also associated with weight loss (*p* = 0.002 for the interaction between time and 24 h α phase). The late phase group (2:40 p.m.–8 p.m.) lost 0.50% ± 0.02% [SE] of initial body weight per week (corresponds to 380 g per week), that was ~1.4 times of the value in the very late phase group (12 a.m.–4 a.m.) (0.37% ± 0.03% per week or 281 g per week; *p* = 0.001). The early phase group lost 0.44% ± 0.02% of initial weight (334 g) per week on average, which was between the values of the other two groups, but the differences from the other two phase groups were not significant (both *p*-values > 0.05).

**Figure 3:**
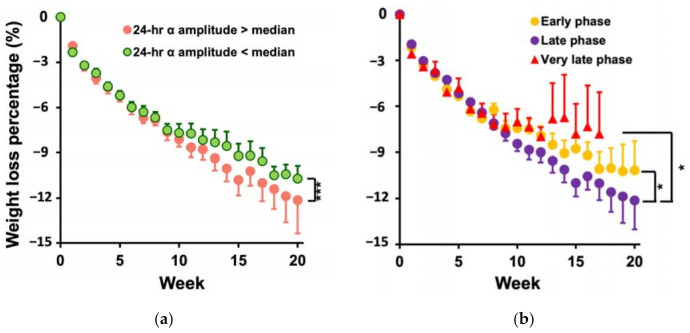
Association of weight loss evolution with the daily rhythm of FCD. Accumulative weight loss in each of the first 20 weeks over the dietary treatment in (**a**) individuals with small (<median) and large amplitudes (>median) and (**b**) individuals with early, late, and very late phases. The results and the *p* value were obtained from the *Model 1b*. Error bars indicate standard errors. * indicates *p* < 0.05, and *** indicates *p* < 0.0001 for the differences in WLS. Results were obtained after adjusting for daily energy intake and physical activity level.

*Model 1b* further revealed that the effects of daily α rhythm were independent from daily energy intake and physical activity level, i.e., after controlling these two factors, an increase of 0.04 (1 SD of all the individual values) in the 24 h α amplitude was associated with an increase of ~0.07% (of the initial body weight) per week in weight loss (*p* = 0.0001, Supplementary Table S1 [48])—an effect about one-sixth of the mean weekly weight loss of all participants (0.45% of the initial weight) that could account for a difference of 1.1 kg in total weight loss during 20 weeks of treatment (mean duration). Figure 3a shows the weight loss evolutions of the high-amplitude group with the 24 h α amplitude >0.088 (median) and the low-amplitude group with the amplitude <0.088. The high-amplitude group lost ~1.3 times more weight (0.11% of initial weight or 84 g) per week than the low-amplitude group (*p* < 0.0001), which accounted for a difference of 1.68 kg in total weight loss during 20 weeks of treatment.

In *Model 1b*, the effect of phase on WLS was also significant (*p* = 0.004); WLS was larger in the late phase group (0.52 ± 0.02% [SE] of initial body weight per week) as compared to the early phase group (0.45± 0.02%, *p* = 0.02) and the very late phase group (0.42 ± 0.03%; *p* = 0.02). Figure 3b shows the three phase-group averages of weight loss evolution during the treatment. The difference in WLS between the late phase and very late phase group (76 g more per week) could lead to a difference of 1.52 kg in total weight loss in 20 weeks of treatment. Similarly, the early phase group lost less weight (53 g less per week, equals to 1.1 kg after the 20 weeks of treatment) as compared to the late phase group. Daily energy intake had no significant contribution to weight loss progression (*p* = 0.09), while physical activity level contributed independently to WLS, i.e., for a decrease of 38 metabolic units (METs) (1 SD of all the individuals) per day per body weight (kg) in physical activity level, weekly weight loss was reduced by 0.12% of initial weight (*p* < 0.0001), which was equivalent to 91 g more per week and could lead to a difference of 1.82 kg in total weight loss in 20 wks.

### 3.4. Genetic Effects on WLS, 24 h α Rhythm, and Their Association

**Figure 4:**
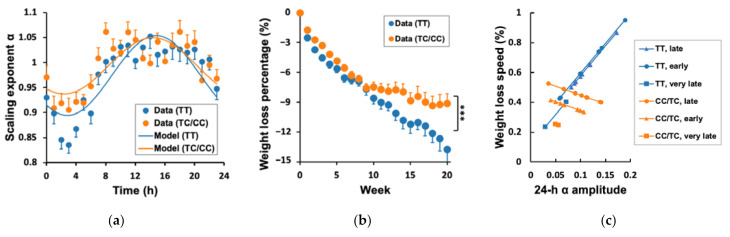
Genetic effects of *CLOCK* 3111 on weight loss speed: (**a**) 24 h α rhythm; (**b**) weight loss evolution; *** indicates *p* < 0.0001 for the difference in WLS between C and TT carriers obtained from *Model 2b* in which the effects of the 24 h α rhythm were controlled; (**c**) The interaction effect of the 24 h α rhythm (amplitude and phase) and *CLOCK* 3111T/C on WLS. The results were obtained from *Model* *3* in which the effects of the phase and amplitude as well as their interaction with the genotype were considered. Weight loss was presented as the percentage of initial body weight. Error bars indicate standard errors.

The group average of the profile of the daily α rhythm was similar between the C carriers and TT carriers (i.e., smaller α during the night-time and larger α during the daytime, Figure 4a). The mixed model using the pooled individual hourly data showed that the amplitude of the rhythm was much smaller in the C group (0.06 ± 0.01) than that in TT carriers (0.08 ± 0.01, *p* < 0.0001). The same model also revealed a slight but significant difference in the phase of the 24 h rhythm, i.e., the C carriers have an earlier peak (2:25 p.m. ± 21 min) than the TT group (2:50 p.m. ± 16 min, *p* < 0.0001). These genetic group differences in the daily α rhythm could not reach a significant level when directly using individual amplitude (*p* = 0.10) and phase values (*p* = 0.33), likely due to insufficient power. Note that, after excluding the three subjects without significant 24 h α rhythms, the number of subjects in three phase groups were the same in the C group and in the TT group (late phase: eight C and eight TT carriers; very late phase: two C and two TT carriers; early phase: eight C and eight TT carriers).

Consistent with previous studies [21], *Model 2a* showed that C carriers had lower WLS as compared to TT carriers (0.10 ± 0.02% [SE] less in C carriers, *p* < 0.0001); Figure 4b). This reduced weight loss efficiency in the C carriers is consistent with the overall of smaller α rhythm (Figure 4). Intriguingly, *Model 2b* showed that the genetic effect on WLS persisted (0.15% ± 0.02% per week in TT carriers) and was not reduced after adjusting for the effects of the amplitude and phase. Specifically, the group average of the weekly weight loss was 1.43 times larger in the T group (0.50% ± 0.02%) than that in the C group (0.35% ± 0.02%, *p* < 0.0001) (Figure 4c), which is equivalent to a 114 g difference in weight loss per week, and 2.28 kg in 20 weeks.

We further explored the potential effect of *CLOCK* 3111T/C on the association between the daily α rhythm and WLS. *Model 3* revealed significant differences between C carriers and T carriers in the effects of both amplitude and phase of daily 24 h α rhythm on the WLS (*p* < 0.0001 for the *CLOCK* * amplitude * week, and *p* = 0.036 for the *CLOCK* * phase * week) (Figure 4c). Specifically, the WLS within C carriers was significantly larger in the late phase group (0.46 ± 0.03% [SE] of initial body weight per week) than that of the early (0.36 ± 0.03%, *p* = 0.03) or very late phase (0.21 ± 0.07% of initial body weight per week) (*p* = 0.004), while TT carriers showed no significant differences in WLS between different phase groups (early phase group: 0.56 ± 0.04%; late phase group: 0.54 ± 0.02%; very late phase group: 0.48 ± 0.03%; all *p* > 0.1 Supplementary Table S1 [48]). In contrast to the phase effect, the association between the 24 h α amplitude and WLS was stronger in TT carriers as compared that in C carries (*p* < 0.001; Figure 4c and Figure S2 [48]), i.e., an increase of 0.04 in the 24 h α amplitude (1 SD of all the individual values) was associated with an increase of 0.14% in WLS within TT carriers but was associated with −0.05% in WLS within C carriers. The genetic effects on the amplitude–WLS and phase–WLS associations were further confirmed by performing statistical models within C carriers and within TT carriers, separately, i.e., the amplitude–WLS association was significant within TT carriers (*p* < 0.0001) but was not significant within C carriers (*p* = 0.08); the phase–WLS association was only significant in the C carrier data (*p* = 0.008) but not in TT carriers (*p* = 0.20). After controlling the effect of the 24 h α rhythm and its interaction with the genotype, the C carriers still had significant lower WLS than the TT carriers (*p* < 0.0001).

## 4. Discussion

This study provides the first evidence that the daily rhythm of fractal cardiac dynamics is linked to the efficacy of weight loss treatment. We found a robust daily rhythm in fractal cardiac dynamics. The phase and amplitude of the 24 h fractal rhythm showed a high inter-individual variability. Intriguingly, both the phase and amplitude of the rhythm were associated with weight loss speed. Importantly, all these associations were independent of daily energy intake and physical activity levels. More interestingly, *CLOCK* 3111 genotype affected the associations of phase and amplitude with weight loss response. Specifically, the association between amplitude and weight loss response was more pronounced in TT carriers, whereas the association between phase and weight loss response was more pronounced in C carriers.

### 4.1. Health Implications

**Figure 5:**
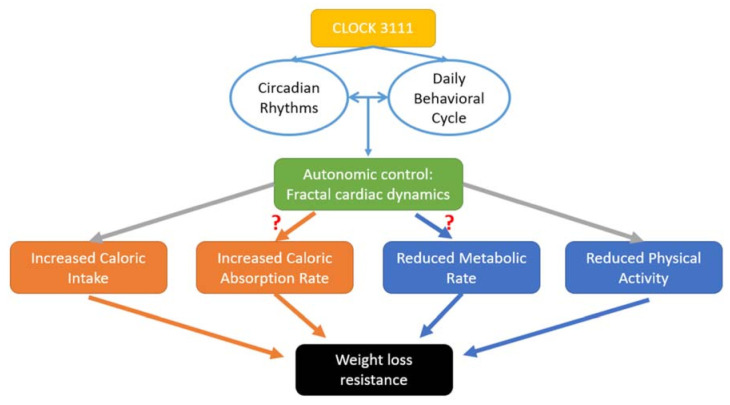
Hypothesized pathways for the genetic influences on weight loss resistance.

Our findings have many health-related implications, especially in today’s society with the obesity epidemic. Many dietary and exercise weight loss treatments work well for some people, while the same treatments have little effects on other people. Little is known about relevant factors affecting weight loss effectiveness, and a large scientific gap remains to be addressed. Previous studies have considered many factors contributing to differences in weight loss effectiveness including the type of diet [4], exercise level [49], and emotional factors [5]. Nutritionists become more and more interested in adopting additional techniques that may complement the core nutritional advice in order to achieve better weight loss. In the women of this study, we show that the daily rhythm of fractal cardiac dynamics is associated with the weight loss response, independent of previously known factors such as energy intake and physical activity level. Alterations or losses of fractal patterns in physiological fluctuations are observed under different pathological conditions such as cardiac diseases [28]. The current data suggest that the inter-individual differences in fractal cardiac dynamics may also potentially provide a better understanding of inter-individual variability in the response to obesity treatment. One possible explanation for our results is the role of the autonomic control in the balance between energy storage and consumption [25] (Figure 5). Since the effects of the daily α rhythm persisted after controlling for daily energy intake and physical activity levels, further studies are required to determine whether the autonomic system is linked to weight loss through the other pathways such as reduced energy expenditure, caloric absorption rate, or changes in adipose tissue accumulation or mobilization due to alterations in the signalling pathways in brown and white adipose tissues. For instance, recent studies showed the evidence that the circadian clock affects adipose tissue metabolism via its influences on differentiation, adipogenesis, and lipolysis, or thermogenic effect through changes in uncoupling proteins (UCP) [50]. Thus, the investigation of fractal cardiac dynamics (a measure of autonomic control) may improve the mechanistic understanding of the weight loss resistance in certain individuals such as *CLOCK* 3111C carriers, which will help develop novel strategies for body weight control. 

### 4.2. Direction of the Relationship between Daily Rhythm of Fractal Cardiac Dynamics and Weight Loss

There might be two different explanations for the observed associations between weight loss response and the characteristics of the daily rhythm of fractal cardiac dynamics. One interpretation is that inter-individual differences in the daily rhythm were already present before the treatment, and such differences predicted or caused different physiological responses to the same dietary–behavioural treatment, leading to different weight loss speed. Alternatively, the observed associations may be caused by the effect of weight loss on the daily rhythm of autonomic function, while the daily rhythm was not or less different before the treatment. This is possible, considering that (1) there are elaborate feedback mechanisms from the body’s energy stores to the hypothalamic centres through the neuroendocrine system [51], and (2) the suprachiasmatic nucleus in the hypothalamus is the central circadian clock that connects to both sympathetic and parasympathetic branches of the autonomic nervous system (ANS), balances their outputs to peripheral organs, and generates/orchestrates the rhythms of ~24 h in ANS activities [52,53]. However, no studies have shown the direct effect of weight loss on the circadian rhythms, especially if the effect depends on the genetic variant *CLOCK* 3111T/C (i.e., we found that weight loss was linked to the phase of the rhythm in C carriers but not in TT carriers, while weight loss was linked to the amplitude of the rhythm in TT carriers but not in C carriers). If this is the case, our results may provide evidence for the pathway through which obesity treatment impacts cardiometabolic health. Based on the second possibility, one logical hypothesis is that body weight was associated with the daily rhythm of fractal cardiac dynamics (if their changes were associated). However, we found no significant associations of body weight after the treatment with the phase or amplitude of the daily rhythm of fractal cardiac dynamics (see Supplementary Table S2 [48]). These results appear not to support the causal effect of body weight changes on the daily rhythm of autonomic function. To formally determine the direction of the relationship between daily rhythm of fractal cardiac dynamics and weight loss, ECG assessment should be performed before and after the treatment.

### 4.3. Optimal Circadian Alignment

One crucial follow-up question is, “what causes the inter-individual variation in the daily rhythm of fractal cardiac dynamics or autonomic control?” One of the most likely candidates is the circadian control system, which is known to have a direct influence on autonomic control as assessed by fractal cardiac dynamics [22]. Indeed, the phase of a daily physiological rhythm may indicate the degree of circadian (mis)alignment that can be affected by both the intrinsic circadian clock, the timing of daily behaviours, and their interaction (Figure 5). One of the well-known examples for adverse health consequences of circadian disruption is shift workers. Overwhelming evidence shows higher obesity levels and disrupted daily rhythm of autonomic function in this population [54,55] who have mistimed daily behaviours. In this study, subjects did not have shift work. Thus, the inter-individual differences in the daily fractal rhythm and their associations with weight loss effectiveness suggest that circadian alignment/misalignment is not an all-or-none phenomenon, and there may exist an “optimal” alignment between the circadian clock and the daily behavioural cycle (e.g., timing of food and exercise) in term of weight loss effectiveness.

An unexpected finding is that, after controlling for energy intake and expenditure, those subjects with the later phase of the α rhythm (2:40 p.m.–8 p.m.) had better weight loss response as compared to those with the phase at earlier times (10 a.m.–2:40 p.m.). Note that the phase of the daily α rhythm was not significantly associated with bedtime (phase: *p* = 0.71; amplitude: *p* = 0.98) or wakeup time (phase: *p* = 0.09; amplitude: *p* = 0.50). Thus, the sleep–wake schedules could not explain the inter-individual variability of the phase. A potential interpretation for these phase effects may be related to social jet lag, e.g., those “evening type” persons might have to wake up earlier, making them imposed to a forced advance in phase (against their circadian preference) due to work schedules or traditional paradigm (sleep and wake up early). Thus, their daily rhythm of behaviours and autonomic function were not optimally aligned, inducing weight loss resistance. Furthermore, supporting the optimal phase concept, we found that it was also difficult for those with very extremely late phase (e.g., after midnight 12 a.m.) to lose weight. Only 30 subjects in the study had the morning–evening score (based on the morning–evening questionnaire) that was not significantly associated with the phase (*p* = 0.5). Future studies with better design, such as interventional studies manipulating the timing of daily behaviours (such as meal, exercise, and sleep) while monitoring energy intake/expenditure and weight loss, are warranted to test the hypothesis about the social jet lag and the optimal phase for weight loss response.

To make the picture more complicated, altered circadian control can affect the daily rhythm of fractal cardiac dynamics (and, thus, weight loss efficiency) directly or indirectly through its influence on or interaction with sleep regulation. Previous studies showed that more sleep fragmentation predicts a lower magnitude of weight loss in overweight and obese women participating in a weight loss intervention [56]. We confirmed this by examining the fractal cardiac patterns and polysomnography-based sleep dynamics in 64 healthy subjects from Sleep Heart Health Study database of the National Sleep Research Resource [57]. We found that the increased fractal cardiac correlations during the night-time sleep (when fractal correlations should be low) were associated with more arousals and more fragmented sleep (*p* < 0.001) and more sleep–wake transitions, *p* = 0.02, Figure S3 [48]). These results support the hypothesis that bad sleep quality underlies the reduced daily rhythm amplitude (higher night-time α value and therefore smaller difference from the daytime value), leading to weight loss resistance. However, this sleep hypothesis cannot explain the observed effect of phase on weight loss, because the phase was not significantly correlated with the 24 h amplitude.

### 4.4. Genetics of Weight Loss Resistance

Though there is overwhelming evidence for the role of genetics in developing obesity [58,59], few studies have examined the genetic basis of weight loss resistance in overweight/obesity persons. In a study of 1495 overweight/obese individuals who participated in the ONTIME project, we previously showed that *CLOCK* 3111C carriers have more difficulty in losing weight than non-carriers [60]. This finding was also supported by other groups [61,62,63]. By monitoring cardiac activity of 20 *CLOCK* 3111C carriers and 19 TT carriers from the ONTIME project, this study was designed to test whether *CLOCK* 3111C carriers have different daily rhythms of fractal cardiac dynamics (reflecting different circadian regulation of autonomic function) and whether the difference is linked to weight loss resistance in these individuals. Our results showed that the 20 *CLOCK* 3111C carriers had suppressed daily rhythms of fractal cardiac dynamics (i.e., lower 24 h amplitude) that were associated with lower weight loss speed. However, the altered daily rhythms in C carriers appeared not able to fully explain the genetic effect on weight loss response. Indeed, after adjusting for the phase and amplitude of the rhythm, the mean effect size for the genetic influence on WLS was not diminished or even increased. Thus, *CLOCK* 3111T/C genotype may impact weight loss response through other pathways (different from the circadian regulation of autonomic function). Furthermore, we found that the *CLOCK* 3111T/C genotype affected the relationship between the daily rhythms of fractal cardiac dynamics and weight loss response. Specifically, the phase of the rhythm was more critical for weight loss among C carriers than among TT carriers, and the amplitude was more critical for TT carriers but had no significant effect on the weight loss speed among C carriers. These results suggested complex interactions between genetics, the circadian regulation (likely linked to the alignment between the circadian rhythm and daily behavioural cycle), autonomic function, and body weight control (Figure 5).

### 4.5. Limitations

This is a pilot interventional study with a weekly assessment of weight for ~20 weeks and a continuous monitoring of cardiac activity for up 3.5 days. Despite the robust findings, there are some limitations to be considered for interpretations. 

One limitation is that ECG assessment in this pilot study was only performed after the weight loss treatment. As a result, we could not distinguish whether the observed inter-individual differences in the daily rhythm of autonomic function led to or were caused by different weight loss response. To address this, we will propose a follow-up study in which weight body and ECG measures will be assessed before, during and immediately after the weight loss treatment and a few months after the treatment. The multiple assessments will also help to determine whether the daily rhythm of autonomic function predicts weight loss, how the rhythm changes across the treatment, and whether the changes in the rhythm are acute or long term.

The other limitation is that only autonomic cardiac control was assessed by examining ambulatory ECG signals. Though this approach (using heartrate variability as a proxy of autonomic function) is typical in field studies, because the assessment is non-invasive, it is worth noting that the autonomic inputs to different organs/systems (such as the musculoskeletal system and the gastrointestinal system) can be different. For instance, during the active period, the autonomic input to blood vessels in the musculoskeletal system helps to increase the blood supply in the movement apparatus, while the autonomic input to blood vessels in the gastrointestinal system helps to decrease the blood supply in the digestive apparatus; these different autonomic inputs are simultaneously regulated by the interaction between the circadian system and daily behavioural cycle [64]. Clearly, it is important to examine whether the daily rhythms of autonomic outputs to other organs/systems are linked to or are better associated with weight loss. Examining these different autonomic outputs may also help identify the specific pathway(s) through which the circadian system impacts weight loss (Figure 5).

In addition, the sample size is small, such that some observed negative associations (e.g., the correlation of phase and weight loss efficiency is not significant in the TT carriers) may be due to the lack of power. Relatedly, few participants had significant weight loss resistance in this study (only three participants lost less than 5% of their initial body weight), which might lead to an underestimated effect of daily fractal rhythm on weight loss. All subjects were women, and it is to be confirmed whether our findings remain applicable for men. Furthermore, this is a field study in which subjects underwent their normal daily activities, and the environmental conditions were not controlled, so it was not possible to assess the endogenous circadian rhythm. Future studies of larger populations with both females and males, with more specific circadian assessment, and with repeated ECG assessments are required to validate our findings and to elucidate the underlying mechanisms.

## 5. Conclusions

This study provides the first evidence that daily rhythm of fractal cardiac dynamics is linked to the efficacy of obesity treatments, independent of energy intake and physical activity, while depending on genetics. Results highlight the complex interactions between autonomic function, circadian regulation, and body weight control. Follow-up studies are encouraged to test whether manipulating the different features of the rhythm (i.e., phase and amplitude) may have different effect sizes for different *CLOCK* 3111 genotypes. Considering the various influences of the circadian regulation and autonomic function on metabolism and cardiovascular physiology, the current and follow-up studies shall have potentially deep impacts on not only obesity treatment but also cardiometabolic health research. In addition, the proposed fractal cardiac measures may serve as a promising tool in clinical settings for assessment or evaluation of obesity treatment. Current results may help researchers understand how timings of daily behaviours such as meal, exercise, and sleep affect the daily rhythm of autonomic function and weight loss. After confirming the optimal timings/phases of these behaviours, we can design individualized weight loss interventions by recommending the best daily schedule in terms of weight loss. New personalized obesity interventions should be based on not only nutrition but also individual circadian characteristics, manipulation of daily schedules, and autonomic function.

## Figures and Tables

**Table 1:** General characteristics of the study population. Data are presented as mean ± SD. NS indicates *p* > 0.1. * One subject (TT carrier) did not complete the weight loss program and, thus, was excluded for the analysis related to weight loss.

	*CLOCK* 3111T/C		
	Risk Carriers (TC + CC)	Non-Risk Carriers (TT)	Group Difference
	n = 20	n = 20	
Obesity Parameters	Mean ± SD	Mean ± SD	*p*-Value
Age (y)	42.0 ± 9.7	46.5 ± 11.04	NS
Height (m)	1.63 ± 0.06	1.63 ± 0.05	NS
Weight (kg)	74.87 ± 8.83	76.32 ± 12.00	NS
BMI (kg/m^2^)	28.14 ± 2.41	28.66 ± 4.41	NS
Waist (cm)	89.10 ± 9.55	91.60 ± 10.80	NS
Hip (cm)	105.65 ± 8.72	110.25 ± 10.50	NS
Body Fat (%)	35.03 ± 3.72	34.81 ± 6.26	NS
Glucose (mg/dL)	84.80 ± 20.47	87.11 ± 10.16	NS
Insulin (µUI/mL)	10.27 ± 13.99	6.03 ± 3.16	NS
Total Triglycerides (mg/dL)	104.35 ± 43.25	87.00 ± 28.90	NS
Total Cholesterol (mg/dL)	198.10 ± 24.67	194.83 ± 26.08	NS
Cholesterol HDL (mg/dL)	57.80 ± 12.99	54.33 ± 14.36	NS
Cholesterol LDL (mg/dL)	119.45 ± 18.65	123.03 ± 26.90	NS
Leptin (ng/mL)	21.14 ± 9.65	20.34 ± 9.32	NS
Adiponectin (ng/mL)	74.60 ± 26.62	78.70 ± 25.24	NS
IL6 (µg/mL)	24.79 ± 5.90	24.81 ± 6.18	NS
Total Energy Intake (kcal/day)	1980 ± 767	1893 ± 601	NS
Physical Activity Test (METs)	3614 ± 2802	2842 ± 2886	NS
Sleep Duration (hours)	7.8 ± 0.6	7.4 ± 0.8	NS
Bedtime	0:36 ± 55 (min)	0:21 ± 47 (min)	NS
Wake-Up Time	8:18 ± 46 (min)	7:47 ± 38 (min)	0.028
Breakfast Time	9:30 ± 64 (min)	8:35 ± 48 (min)	0.004
Lunch Time	14:51 ± 29 (min)	14:20 ± 23 (min)	NS
Dinner Time	21:32 ± 32 (min)	21:25 ± 24 (min)	NS
Intervention Duration (Weeks)	16.9 ± 6.6	16.8 ± 9.4 *	NS
Total Weight Loss (Kg)	7.18 ± 2.20	9.02 ± 4.56 *	NS
Medication Use (Total)	9	9	NS
Anti-hypertensives	2	2	NS
Lipid Lowering Drug	1	1	NS
Anti-diabetics	0	1	NS
Antidepressants	3	4	NS
Anxiolytics	2	3	NS
Thyroid Hormone	3	0	0.036
Contraceptives	1	3	NS

## Data Availability

Some or all datasets generated during and/or analyzed during the current study are not publicly available but are available from the corresponding author on reasonable request.

## Supplementary Materials

The following are available online at https://www.mdpi.com/article/10.3390/nu13072463/s1, Figure S1: Inter-individual variabilities in the daily rhythm of fractal cardiac dynamics. Figure S2: Difference between C and T groups in the association of weekly weight loss % and the amplitude of 24 hr α rhythm. Figure S3. Associations between fractal cardiac correlations, indicated by scaling exponent α, and sleep dynamics. Table S1: Summary statistics for all the models. Table S2: Correlation between final weight and 24 h alpha.
