# Supplementary material for: Daily Rhythm of Fractal Cardiac Dynamics Links to Weight Loss Resistance: Interaction with CLOCK 3111T/C Genetic Variant
Source: Nutrients. 2021 Jul 19;13(7):2463. doi: 10.3390/nu13072463 (PMC8308644; doi:10.3390/nu13072463)
Supplement: Supplementary file 1 [file nutrients-13-02463-s001.zip › nutrients-1276157-supplementary.pdf]

Supplementary Figures

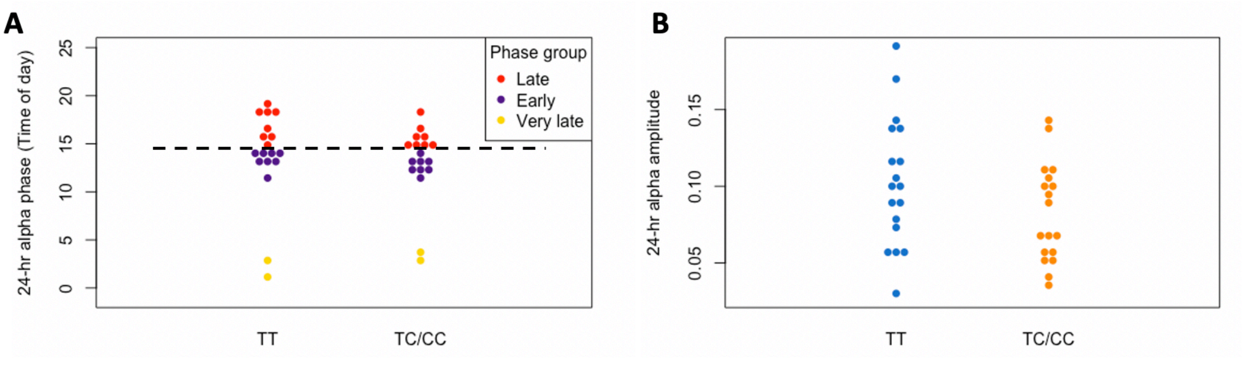

Figure S1. **Inter-individual variabilities in the daily rhythm of fractal cardiac dynamics.** (**A**) Distribution of the of phase of the 24-h α rhythm in the TT carriers and TC/CC carriers. (**B**) Distribution of the amplitude of the 24-h α rhythm in the TT carriers and TC/CC carriers. The dashed line indicates median value of the phase of the majority (the peak of the 24-h component) at 14:40. These subjects were further divided into three groups: early phase (10 a.m. - 2:40 p.m.), late phase (2:40 p.m. – 8 p.m.) and very late (>12 a.m.).

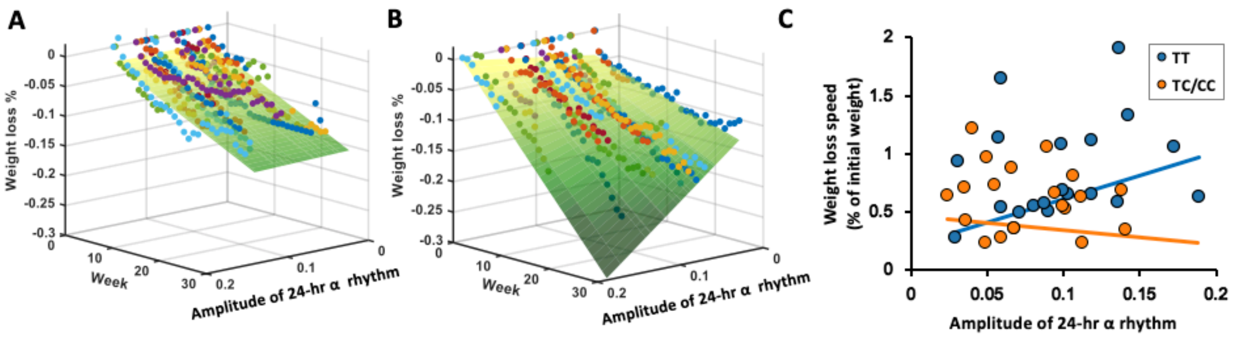

Figure S2. **Difference between C and T groups in the association of weekly weight loss % and the amplitude of 24-hr α rhythm**. (**A-B)** Weight loss for different 24-h amplitude over the 20 weeks in C carriers (**A**) and T carriers (**B**). Dots of the same color indicate record from one individual. The surface is the predicted value from our model; the more value of weight loss is indicated by the deeper color. (**C**) Mean individual weekly weight loss percentage (WLS) for different 24-h α amplitude. Weight loss was presented as the percentage of initial body weight.

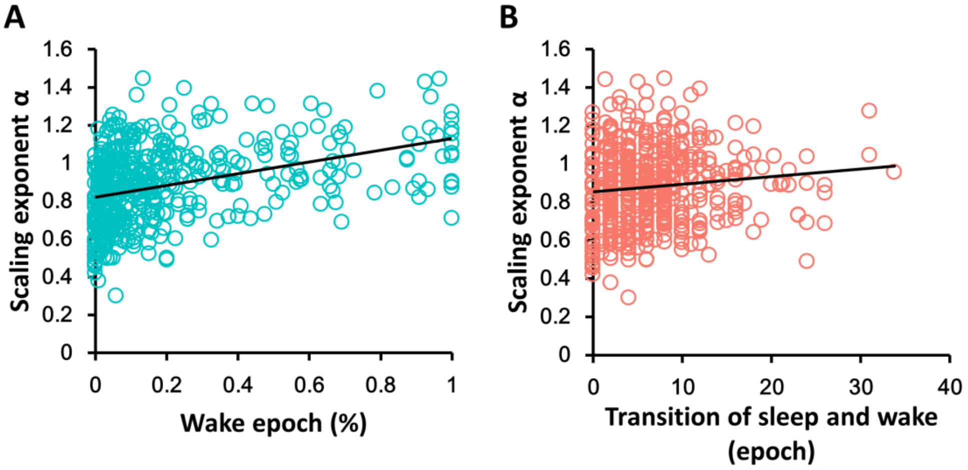

Figure S3. Associations between fractal cardiac correlations, indicated by scaling exponent α, and sleep dynamics. (**A**) Correlation between proportion of epochs that were labeled as wakefulness and the fractal cardiac correlations. Solid line represents significant a linear correlation with R^2=0.14, p<0.001. (**B**) Correlation between number of epochs that change from sleep to wakefulness or wakefulness to sleep. Solid line represents a significant linear correlation with R^2=0.01 and p=0.02. Sleep polysomnography data were collected from 64 subjects. Sleep state (wakefulness or sleep) is labeled on each 30-sec epoch. The fractal cardiac correlations and sleep dynamics are analyzed in each 1-hour segment, and a total of 488 hours were used.

Supplementary Table S1: Summary statistics for all the models.

Model 1a: Time, amplitude and phase

Type III Analysis of Variance Table with Satterthwaite's method

	Sum Sq	Mean Sq	NumDF	DenDF	F value	P-value	
Time	3867.1	3867.1	1	620.32	1220.64	<0.0001	***
Amp24	1.5	1.5	1	39.98	0.47	0.4957	
phase24_3group	0.7	0.4	2	38.27	0.11	0.8943	
Time:Amp24	37.3	37.3	1	625.46	11.77	0.0006	***
Time:phase24_3group	41.6	20.8	2	621.41	6.56	0.0015	**

Model 1a: post hoc

Effect of Phase on Time	Estimate	SE	lower 95% CI	upper 95% CI	P-value Compare with Early phase	P-value Compare with Late phase
Very late phase	0.371	0.029	0.314	0.428	0.0782	0.0010
Early phase	0.444	0.022	0.401	0.486		0.1142
Late phsae	0.499	0.016	0.468	0.529		

Effect of Amplitude on Time	Estimate	SE	t val.	P-value
Slope of Time when Amplitude = -1SD	0.393	0.022	17.053	<0.0001
Slope of Time when Amplitude = mean	0.444	0.022	20.666	<0.0001
Slope of Time when Amplitude = +1SD	0.495	0.029	17.108	<0.0001

Effect of Amplitude on Time (2 groups)	Estimate	SE	lower 95% CI	upper 95% CI	P-value Compare with < median
Slope of Time when Amplitude > median	0.485	0.021	0.444	0.527	0.0002
Slope of Time when Amplitude < median	0.393	0.014	0.366	0.419	

Model 1b: Time, amplitude and phase, including MET and energy intake

Type III Analysis of Variance Table with Satterthwaite's method

	Sum Sq	Mean Sq	NumDF	DenDF	F value	P-value	
Time	2846.4	2846.4	1	572.99	927.29	<0.0001	***
Amp24	0.3	0.3	1	32.77	0.10	0.7561	
phase24_3group	1.62	0.81	2	31.76	0.26	0.7697	
MET_normal	2.15	2.15	1	33.65	0.70	0.4084	
TotalEnrIn_normal	1.95	1.95	1	33.98	0.63	0.4314	
Time:Amp24	57.01	57.01	1	576.80	18.57	<0.0001	***
Time:phase24_3group	34.02	17.01	2	572.36	5.54	0.0041	**
Time:MET	102.55	102.55	1	566.85	33.41	<0.0001	***
Time:TotalEnergyIntake	8.63	8.63	1	577.00	2.81	0.0942	.

Model 1b: post hoc

Effect of Phase on Time	Estimate	SE	lower 95% CI	upper 95% CI	P-value Compare with Early phase	P-value Compare with Late phase
Very late phase	0.417	0.034	0.350	0.484	0.7461	0.0159
Early phase	0.446	0.022	0.402	0.490		0.0226
Late phsae	0.522	0.016	0.491	0.553		

Effect of Amplitude on Time	Estimate	SE	t val.	P
Slope of Time when Amplitude = -1SD	0.377	0.023	16.758	<0.0001
Slope of Time when Amplitude = mean	0.446	0.022	20.019	<0.0001
Slope of Time when Amplitude = +1SD	0.514	0.032	16.347	<0.0001

Effect of Amplitude on Time (2 groups)	Estimate	SE	lower 95% CI	upper 95% CI	P-value Compare with < median
Slope of Time when Amplitude > median	0.514	0.022	0.471	0.557	<0.0001
Slope of Time when Amplitude < median	0.409	0.016	0.378	0.440	

Model 2a: CLOCK 3111 genetic variant and time

Type III Analysis of Variance Table with Satterthwaite's method

	Sum Sq	Mean Sq	NumDF	DenDF	F value	P-value	
Time	5308.8	5308.8	1	625.14	1551	<0.0001	***
CLOCKTC	0.7	0.7	1	43.02	0.2004	0.6567	
Time:CLOCKTC	60.6	60.6	1	625.14	17.698	<0.0001	***

Model 2a: post hoc

Effect of CLOCK on Time	Estimate	SE	lower 95% CI	upper 95% CI	P-value Compare with CC/CT
TT	0.395	0.027	0.362	0.429	<0.0001
CC/CT	0.490	-0.015	0.461	0.518	

Model 2b: CLOCK, time, phase and amplitude

Type III Analysis of Variance Table with Satterthwaite's method

	Sum Sq	Mean Sq	NumDF	DenDF	F value	P-value	
Time	3617.4	3617.4	1	619.11	1212.67	<0.0001	***
Amp24	4.00	4.0	1	38.78	1.34	0.2542	
CLOCKTC	0.00	0.0	1	38.04	0.00	0.9840	
phase24_3group	1.30	0.6	2	37.6	0.21	0.8108	
Time:Amp24	47.00	47.0	1	624.61	15.75	<0.0001	***
Time:CLOCKTC	119.20	119.2	1	620.54	39.98	<0.0001	***
Time:phase24_3group	76.70	38.4	2	621.82	12.86	<0.0001	***

Model 2b: post hoc

Effect of Phase on Time	Estimate	SE	lower 95% CI	upper 95% CI	P-value Compare with Early phase	P-value Compare with Late phase
Very late phase	0.319	0.029	0.261	0.376	<0.0001	<0.0001
Early phase	0.490	0.022	0.446	0.533		0.863
Late phase	0.475	0.016	0.444	0.505		

Effect of Amplitude on Time	Estimate	SE	t val.	P-value
Slope of Time when Amplitude = -1SD	0.433	0.023	18.683	<0.0001
Slope of Time when Amplitude = mean	0.490	0.022	22.288	<0.0001
Slope of Time when Amplitude = +1SD	0.548	0.029	18.778	<0.0001

Effect of CLOCK on Time	Estimate	SE	t val.	P-value	P-value Compare with CC/CT
TT	0.503	0.016	0.471	0.535	<0.0001
CC/CT	0.352	0.018	0.316	0.388	

Model 3: Interaction of CLOCK, time, amplitude and phase

Type III Analysis of Variance Table with Satterthwaite's method

	Sum Sq	Mean Sq	NumDF	DenDF	F value	P-value	
Time	2258.52	2258.52	1	610.69	802.73	<0.0001	***
Amp24	1.95	1.95	1	34.22	0.69	0.4113	
CLOCKTC	0.09	0.09	1	33.48	0.03	0.8561	
phase24_3group	0.96	0.48	2	34.17	0.17	0.8443	
Time:Amp24	30.78	30.78	1	614.59	10.94	<0.0001	***

Time:CLOCKTC	88.89	88.89	1	610.69	31.59	<0.0001	***
Amp24:CLOCKTC	2.95	2.95	1	34.22	1.05	0.3134	
Time:phase24_3group	35.58	17.79	2	612.81	6.32	0.0019	**
CLOCKTC:phase24_3group	0.51	0.26	2	34.17	0.09	0.9134	
Time:CLOCKTC:Amp24	105.38	105.38	1	614.59	37.46	<0.0001	***
Time:CLOCKTC:phase24_3group	18.84	9.42	2	612.81	3.35	0.0358	*

Model 3: post hoc

**Effect of Phase on Time**	**Estimate**	**SE**	**lower 95% CI**	**upper 95% CI**	**P-value Compare with Early phase**	**P-value Compare with Late phase**
**CLOCK = CC/TC**						
Very late phase	0.206	0.069	0.070	0.342	0.0806	0.0038
Early phase	0.356	0.025	0.306	0.406		0.0317
Late phase	0.460	0.027	0.407	0.514		
**CLOCK = TT**						
Very late phase	0.479	0.033	0.413	0.544	0.3626	0.3001
Early phase	0.556	0.043	0.472	0.640		0.9156
Late phase	0.538	0.018	0.503	0.573		

**Effect of Amplitude on Time**	**Estimate**	**SE**	**t val.**	**P-value**
**CLOCK = CC/TC**				
Slope of Time when Amplitude = -1SD	0.398	0.025	16.260	<0.0001
Slope of Time when Amplitude = mean	0.356	0.025	14.091	<0.0001
Slope of Time when Amplitude = +1SD	0.314	0.041	7.729	<0.0001
**CLOCK = TT**				
Slope of Time when Amplitude = -1SD	0.415	0.050	8.262	<0.0001
Slope of Time when Amplitude = mean	0.556	0.043	13.012	<0.0001
Slope of Time when Amplitude = +1SD	0.698	0.044	15.720	<0.0001

Model 3: Subset TT group

Type III Analysis of Variance Table with Satterthwaite's method

	**Sum Sq**	**Mean Sq**	**NumDF**	**DenDF**	**F value**	**P-value**	
Time	2444.99	2444.99	1	309.953	1069.88	<0.0001	***
Amp24	4.47	4.47	1	16.018	1.9544	0.1812	
phase24_3group	0.53	0.26	2	15.493	0.1155	0.8917	
Time:Amp24	134.01	134.01	1	309.937	58.6403	<0.0001	***
Time:phase24_3group	7.36	3.68	2	309.523	1.6093	0.2017	

Model 3: Subset CC/TC group

Type III Analysis of Variance Table with Satterthwaite's method

	**Sum Sq**	**Mean Sq**	**NumDF**	**DenDF**	**F value**	**P-value**	
Time	538.25	538.25	1	301.353	160.64	<0.0001	***
Amp24	0.06	0.06	1	19.27	0.02	0.897998	
phase24_3group	0.87	0.43	2	20.344	0.13	0.879358	
Time:Amp24	10.06	10.06	1	303.637	3.00	0.084207	.
Time:phase24_3group	32.48	16.24	2	300.642	4.85	0.008481	**

Supplementary Table S2: Correlation between Final weight and 24-hr alpha

1. Final weight and 24-hr alpha amplitude

Dependent variable: Final weight (KG)

Independent variable: 24-hr alpha amplitude

Method: linear regression

	Parameter estimates			
	Esitmate	Std Error	t Ratio	P-value
24-hr alpha amplitude (N=39)	-15.43	46.43	-0.33	0.74

2. Final weight and 24-hr alpha phase

Dependent variable: Final weight (KG)

Explanatory variable: 24-hr alpha phase in 3 groups

Method: oneway ANOVA

	Parameter estimates			
	mean	SE	F Ratio	P-value
Early phase (N=16)	72.13	3.01	0.02	0.97
Late phase (N=16)	72.2	3.01		
Very late phase (N=4)	73.5	6.02		
